# Clinicopathological features and surgical treatments of intraductal papillary neoplasm of the bile duct: a case report and literature review

**DOI:** 10.3389/fmed.2024.1443599

**Published:** 2024-09-25

**Authors:** Chang Fu, Hengwei Jin, Yongxin Wang, Hongji Xu

**Affiliations:** ^1^Department of Hepatobiliary and Pancreatic Surgery, General Surgery Center, The First Hospital of Jilin University, Changchun, China; ^2^Department of Abdominal Surgery, Guiqian International General Hospital, Guiyang, China

**Keywords:** intraductal papillary neoplasm of the bile duct, cholangiocarcinoma, hepatobiliary disease, clinical features, prognosis

## Abstract

Intraductal papillary neoplasm of bile duct (IPNB), as a precancerous lesion of cholangiocarcinoma, is a rare biliary tract tumor. A 66-year-old female patient was found to have a bile duct mass by routine examination. The liver function tests and tumor markers were normal. Imaging findings revealed a 2.6 cm mass in the common hepatic duct, accompanied by dilatation of both intrahepatic and extrahepatic bile ducts. The patient underwent open extrahepatic bile duct resection, cholecystectomy and Roux-en-Y hepaticojejunostomy. We also conducted a literature review to summarize the clinicopathological features and surgical treatments of IPNB.

## Introduction

Intraductal papillary neoplasm of bile duct (IPNB) is a rare bile duct tumor, which was first reported by Chen et al. (1). It is characterized by intraductal papillary or villous biliary neoplasms covering delicate fibrovascular stalks (2). According to the 2019 World Health Organization classification of tumors of the digestive system, IPNB is defined as an intraepithelial neoplasm based on its site of origin, excessive mucin secretion, prognosis, and histological features (3). Here, we report a case and summarize previous IPNB cases in the literature, in order to enhance understanding of the clinicopathological features and surgical methods of this rare disease and achieve the goal of early diagnosis and treatment.

## Case presentation

**Figure 1:**
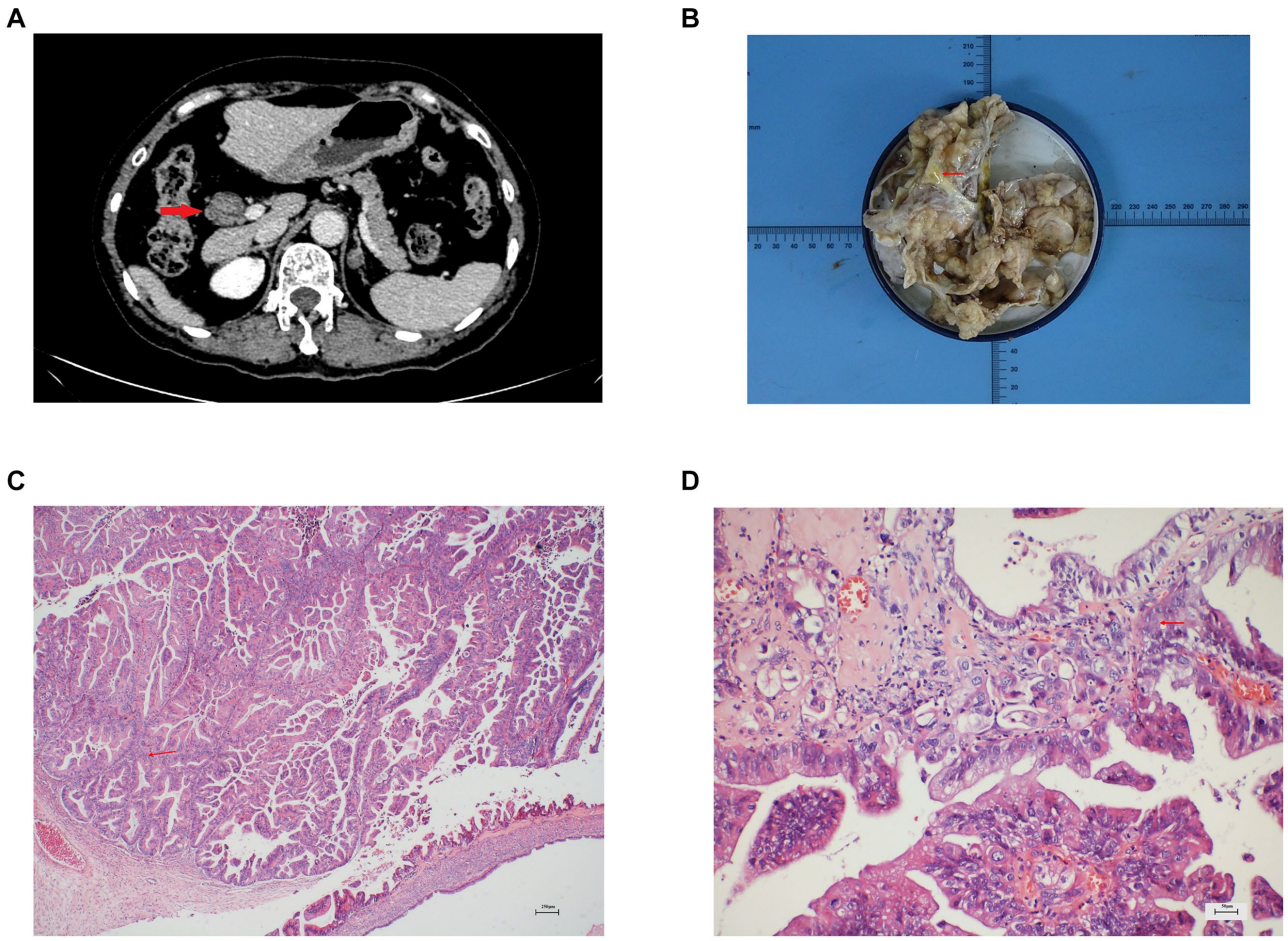
**(A)** Enhanced CT showed a round low-density mass in the hepatic hilar area and dilated bile duct, **(B)** Gross pathology showed the bile duct is filled with greenish-yellow mucus-like sludge, **(C)** Microscopic finding showed the characteristics of intraductal papillary growth (hematoxylin and eosin stain; ×40 original magnification), **(D)** Microscopic finding showed the papillary growth neoplasia with high-grade intraepithelial neoplasia (hematoxylin and eosin stain; ×200 original magnification).

A 66-year-old female patient was admitted because of a mass discovered in the common hepatic duct during routine examination. The patient reported occasional abdominal distension but denied experiencing fever or abdominal pain. Physical examination had no remarkable findings. Enhanced computed tomography (CT) and magnetic resonance cholangiopancreatography (MRCP) showed obvious dilatation of the common bile duct, nodular soft tissue shadow with a diameter of 2.6 cm in the common hepatic duct, and dilatation of the intrahepatic and extrahepatic bile duct (Figure 1A and Supplementary Figures S1–S4). The tumor was located 1 cm below the bifurcation of the bile duct. Laboratory results indicated normal levels of liver enzymes, bilirubin, and tumor markers. Based on the results of laboratory tests and imaging, the preliminary diagnosis was IPNB. During exploration through a right upper abdominal rectus muscle incision, a lesion measuring 2.3 × 2 × 0.8 cm was identified in the common hepatic duct, accompanied by the observation of greenish-yellow mucus-like sludge (Figure 1B). No peripheral enlarged lymph nodes were found during the operation. The patient underwent open extrahepatic bile duct resection, cholecystectomy and Roux-en-Y hepaticojejunostomy. During the operation, negative margins were ensured through intraoperative frozen section diagnosis, and the left and right hepatic ducts were anastomosed to the jejunal loop separately. The operation took 115 minutes with an estimated blood loss of 30 mL. Microscopically, the common bile duct was lined by papillary growth neoplasia with high-grade intraepithelial neoplasia (Figures 1C,D).

Postoperative laboratory tests showed that liver enzymes and bilirubin were normal. The patient recovered well and was discharged after one week. After 18 months of follow-up, the patient had no tumor recurrence.

## Discussion

IPNB, a rare intraductal preinvasive tumor, share characteristics akin to those of intraductal papillary mucinous neoplasm of the pancreas (IPMN). Arising from biliary tree stem cells in the peribiliary glands, IPNB encompasses gastric, oncocytic, pancreaticobiliary, and intestinal subtypes (4). The prevelent pancreaticobiliary subtype in Western population, characterized by the expression of the mucin core protein MUC1, bears a heightened risk of malignant progression. Conversely, the intestinal subtype, prevalent in Asian population and expressing MUC2 and MUC5AC, is more common. In the pancreaticobiliary histological type, the biliary type is more closely associated with malignant transformation, whereas the pancreatic subtype exhibits more mucin production (5). Remarkably, over 50% of IPNB cases coincide with invasive carcinoma (6, 7).

IPNB mostly occurs in East Asia, with some studies indicating its association with the high prevalence of hepatolithiasis and clonorchiasis (8, 9). Chronic biliary inflammation caused by hepatolithiasis and *Clonorchis sinensis* infection leads to the production of reactive oxygen or nitrogen species, which subsequently damage DNA and cause neoplastic changes in the biliary epithelium, ultimately resulting in the development of IPNB (10, 11). Additionally, IPNB exhibits significant early genetic alterations. Mutations in cancer-related genes TP53, KRAS, GNAS, and SMAD4 may lead to the inactivation of histone modifiers, activation of G protein signaling, and loss of genomic stability (12–14). Typically, IPNB is detected in individuals aged between 50 and 70 years, with a slightly higher incidence among males (15). It frequently presents with nonspecific clinical symptoms, and the most common symptoms are abdominal pain, fever and jaundice due to recurrent biliary obstruction. Abnormal liver enzyme and bilirubin levels may result from biliary obstruction. Some studies suggest that tumor markers are often deemed nonspecific for assessing IPNB malignancy, as their values mainly depend on the degree of biliary obstruction (16–18). Imaging examinations such as CT, magnetic resonance imaging (MRI) and ultrasound are commonly used for the diagnosis of IPNB. The typical imaging findings are bile duct dilatation and intraductal masses (5). In addition, the application of direct cholangiography, such as endoscopic retrograde cholangiopancreatography (ERCP), Spyglass, and percutaneous transhepatic cholangiogram (PTC), can improve the accuracy of diagnosis (19, 20).

Surgical resection is the first choice for the treatment of IPNB, which should be determined according to the location and extent of the tumor (21). Intraoperative frozen section is essential to ensure achieving R0 resection (22). For patients ineligible for surgery, endoscopic radiofrequency ablation (ERFA), argon plasma coagulation (APC) and photodynamic therapy (PDT) can be used for local treatment (23–27). Studies indicate that prognostic factors for IPNB include tumor invasiveness, margin status and lymph node metastasis (28–30). Gordon-Weeks et al. reported a 5-year overall survival rate of 65%, which was better than that of cholangiocarcinoma (7).

Due to the low incidence of IPNB and the limited number of large-sample studies, its clinicopathological features have not been fully elucidated. A multicenter retrospective study involving 85 IPNB patients showed a median age of 66 years, with 49.4% being female, and the overall 5-year postoperative survival rate was 63% (31). Kubota et al. focused on the differences in clinicopathological features and prognosis among different subtypes of IPNB (32). Another study conducted in Thailand found that lymph node metastasis and the completeness of resection are important factors affecting the prognosis of IPNB patients (33).

To comprehensively investigate the clinicopathological features and prognosis of IPNB, we conducted a review of case reports published in Pubmed over the past 22 years (Table 1). A total of 58 patients with ages ranging from 22 to 87 years (median age 68.5 years) were included, which 75.8% of the patients were over 60 years of age. There were more male patients than female patients in this literature review. Notably, IPNB demonstrated pronounced geographical distribution differences, with the majority of cases (75.9%) occurring in Asia. Abdominal pain (41.4%) is the most common symptoms, followed by jaundice (27.6%). The maximum diameter of the tumors ranged from 1 to 13 cm (median:3.3 cm). IPNB can occur in any part of the biliary tract, with 69.0% located in the liver, 20.7% in the common bile duct, 8.6% in the hilar of the liver and 1.7% in the extrahepatic duct and liver. Imaging findings often reveal lesions accompanied by bile duct dilatation. Approximately 1/3 of patients have elevated total bilirubin and liver enzymes due to biliary obstruction caused by IPNB. Tumor markers were normal in more than half of the patients. Among those undergoing radical tumor resection, 25 patients underwent hepatectomy, 17 patients underwent hepatectomy with extrahepatic bile duct resection and hepaticojejunostomy, and 2 patients underwent pancreaticoduodenectomy. Nine patients were treated with local treatments, including ERCP (*n* = 5), ERFA (*n* = 2), APC (*n* = 1), and PDT (*n* = 1), because the physical conditions were not suitable for surgery. Three patients underwent hepatectomy combined with distal pancreatectomy (*n* = 2) and hepatectomy combined with pancreaticoduodenectomy (*n* = 1) because of the presence of IPMN. Two patients underwent hepatectomy combined with gastric wedge resection (*n* = 1) and hepatectomy combined with lobectomy of lung (*n* = 1) because of the aggressive nature of IPNB (Table 1). Pathological results showed that IPNB was often aggressive, of which 65.5% showed high-grade intraepithelial neoplasia or cholangiocarcinoma. Immunohistochemistry results revealed positive expression of CK7 (48.3%), MUC5AC (69.0%) and MUC6 (55.2%) in IPNB. The median recurrence-free survival was 14 months (range, 2–52 months). Tumor recurrence or metastasis was found in 8 patients during the follow-up, whose IPNB pathology were high-grade intraepithelial neoplasia or cholangiocarcinoma (Table 2). Among them, three patients received adjuvant chemotherapy, one patient received metastasectomy combined with chemotherapy, one patient received metastasectomy combined with radiotherapy, one patient underwent radical resection, and two patients received symptomatic treatment (Table 2). For patients with recurrence and metastasis, obstruction complications should be actively treated. Due to the limited number of IPNB cases, there is currently no established treatment standard for patients with recurrent tumors. In conclusion, the treatment for malignant IPNB can be formulated by referring to cholangiocarcinoma, and the treatment for recurrent IPNB should be individualized according to the patient’s physical tolerance.

**Table 1:** The demographic characteristics, clinical features, and surgical treatments of IPNB.

Feature	*N* (%)
Age (years)	
20–40	2 (3.4%)
40–60	12 (20.7%)
60–80	35 (60.3%)
>80	9 (15.5%)
Sex	
Male	34 (58.6%)
Female	24 (41.4%)
Region	
Asia	44 (75.9%)
Europe	7 (12.1%)
North America	5 (8.6%)
South America	2 (3.4%)
Tumor size (cm)	
Range	(1.0–13.0)
Median	(3.3)
Symptom	
Abdominal pain	24 (41.4%)
None	17 (29.3%)
Jaundice	16 (27.6%)
Cholangitis	5 (8.6%)
Unknown	3 (5.2%)
Abdominal mass	2 (3.4%)
Nausea	1 (1.7%)
Tumor location	
The right lobe of the liver	18 (31.0%)
The left lobe of the liver	22 (37.9%)
Extrahepatic duct and liver	1 (1.7%)
Common bile duct	12 (20.7%)
Hilar of the liver	5 (8.6%)
Imagine findings	
Cystic lesion	17 (29.3%)
Solid lesion	7 (12.1%)
Cystic-solid lesion	7 (12.1%)
Dilation of the intrahepatic bile duct	23 (39.7%)
Dilation of the extrahepatic bile duct	4 (6.9%)
Intrahepatic and extrahepatic bile duct dilation	15 (25.9%)
Unknown	4 (6.9%)
Tumor markers	
CA19-9	
Elevated	12 (20.7%)
Normal	33 (56.9%)
Unknown	13 (22.4%)
CEA	
Elevated	7 (12.1%)
Normal	31 (53.4%)
Unknown	20 (34.5%)
AFP	
Elevated	0 (0%)
Normal	27 (46.6%)
Unknown	31(53.4%)
TB	
Elevated	22 (37.9%)
Normal	20 (34.5%)
Unknown	16 (27.6%)
ALT	
Elevated	23 (39.7%)
Normal	19 (32.7%)
Unknown	16 (27.6%)
AST	
Elevated	20 (34.5%)
Normal	21 (36.2%)
Unknown	17 (29.3%)
ALP	
Elevated	28 (48.3%)
Normal	18 (31.0%)
Unknown	12 (20.7%)
Surgical treatments	
Hepatectomy	25 (43.1%)
Hepatectomy; extrahepatic bile duct resection; hepaticojejunostomy	17 (29.3%)
Pancreatoduodenectomy	2 (3.4%)
Endoscopic treatment	5 (8.6%)
ERFA, APC and PDT	4 (6.9%)
Hepatectomy; distal pancreatectomy	2 (3.4%)
Hepatectomy; pancreatoduodenectomy	1 (1.7%)
Hepatectomy; lobectomy of lung	1 (1.7%)
Hepatectomy; gastric wedge resection	1 (1.7%)

CA19-9, carbohydrate antigen 19–9; CEA, carcinoembryonic antigen; AFP, alpha fetoprotein; TB, total bilirubin; ALT, alanine aminotransferase; AST, aspartate aminotransferase; ALP, alkaline phosphatase; ERFA, endoscopic radiofrequency ablation; APC, argon plasma coagulation; PDT, photodynamic therapy.

**Table 2:** The pathological features and prognosis of IPNB.

Feature	*N* (%)
Pathological findings	
No invasion	7 (12.1%)
Low grade	8 (13.8%)
Intermediate grade	5 (8.6%)
High grade	15 (25.9%)
IPNB with cholangiocarcinoma	23 (39.7%)
Immunohistochemistry (+)	
CK7	14/29 (48.3%)
CK19	8/29(27.6%)
CK20	5/29 (17.2%)
MUC5AC	20/29 (69.0%)
MUC6	16/29 (55.2%)
MUC1	6/29 (20.7%)
Current status	
Alive	47 (81.0%)
Dead	3 (5.2%)
Unknown	8 (13.8%)
Recurrence	8 (13.8%)
RFS (month)	Range (2–52)
	Median (14)

RFS, Recurrence-free Survival.

In conclusion, as a premalignant lesion of cholangiocarcinoma, IPNB mostly occurs in elderly male patients. The common symptoms of IPNB were abdominal pain and jaundice. Laboratory tests may reveal elevated bilirubin and liver enzymes. The imaging features were lesion and bile duct dilatation. Most IPNB cases are high-grade intraepithelial neoplasia or invasive carcinoma. Therefore, early diagnosis, prompt treatment and regular postoperative follow-up are of great significance for IPNB.

## Data Availability

The original contributions presented in the study are included in the article/Supplementary material, further inquiries can be directed to the corresponding author.

## Supplementary material

SUPPLEMENTARY FIGURES S1–S4 Enhanced CT (axial, coronal, and sagittal views) and MRCP revealed significant dilation of the intrahepatic bile ducts.
