# Supplementary figures and images for: Clinicopathological features and surgical treatments of intraductal papillary neoplasm of the bile duct: a case report and literature review
Source: Front Med (Lausanne). 2024 Sep 25;11:1443599. doi: 10.3389/fmed.2024.1443599 (PMC11461345; doi:10.3389/fmed.2024.1443599)

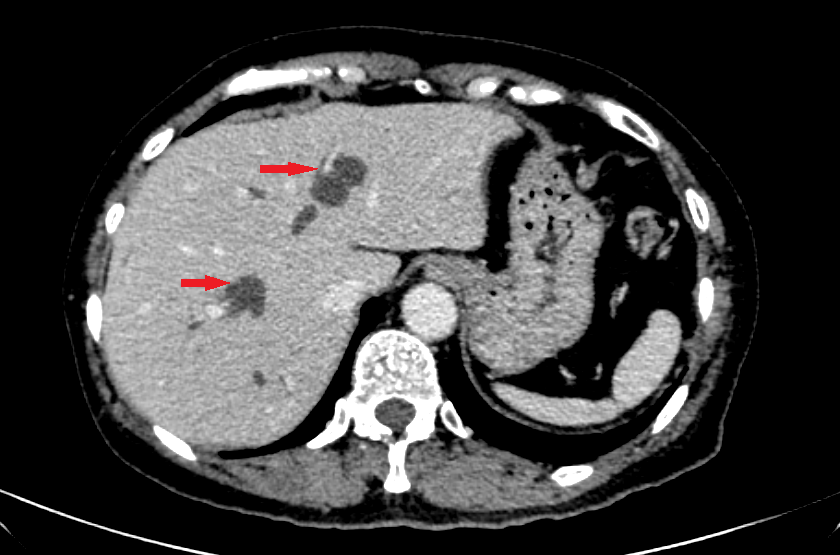

Supplement: SUPPLEMENTARY FIGURES S1–S4 — Enhanced CT (axial, coronal, and sagittal views) and MRCP revealed significant dilation of the intrahepatic bile ducts. [file Image_1.TIF]

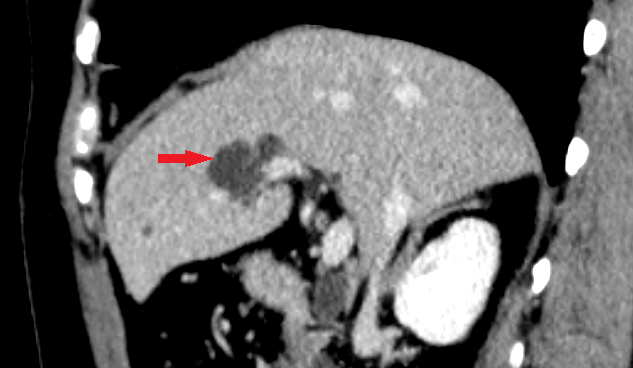

Supplement: Supplementary file 2 [file Image_2.TIF]

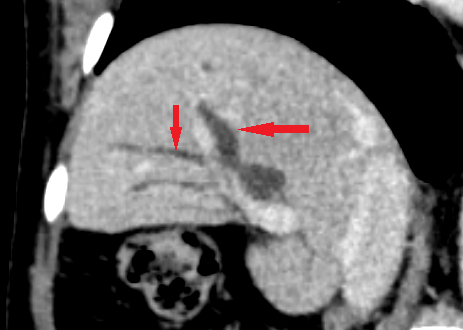

Supplement: Supplementary file 3 [file Image_3.TIF]

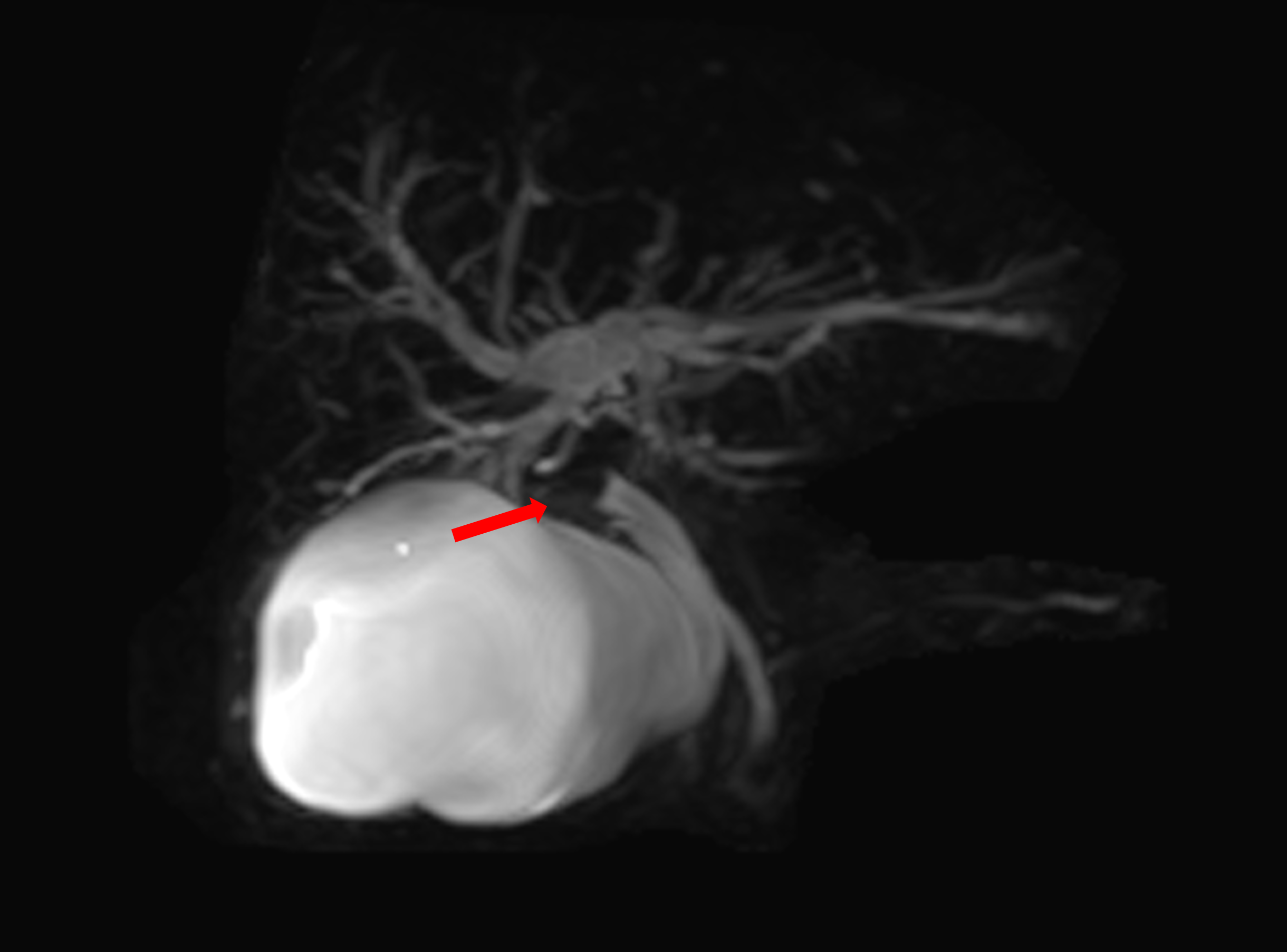

Supplement: Supplementary file 4 [file Image_4.PNG]
